# Fetal Kidney Grafts and Organoids from Microminiature Pigs: Establishing a Protocol for Production and Long-Term Cryopreservation

**DOI:** 10.3390/ijms25094793

**Published:** 2024-04-27

**Authors:** Yuka Inage, Koki Fujimori, Masaki Takasu, Kenji Matsui, Yoshitaka Kinoshita, Keita Morimoto, Nagisa Koda, Shutaro Yamamoto, Kentaro Shimada, Takashi Yokoo, Eiji Kobayashi

**Affiliations:** 1 Division of Nephrology and Hypertension, Department of Internal Medicine, The Jikei University School of Medicine, Tokyo 105-8461, Japan; uzuka@jikei.ac.jp (Y.I.);; 2 Department of Pediatrics, The Jikei University School of Medicine, Tokyo 105-8461, Japan; 3 Sumitomo Pharma, Co., Ltd., Osaka 541-0045, Japan; koki.fujimori@sumitomo-pharma.co.jp (K.F.);; 4 Center for One Medicine Innovative Translational Research (COMIT), Gifu University, Gifu 501-1193, Japan; 5 Department of Urology, Graduate School of Medicine, The University of Tokyo, Tokyo 113-8654, Japan; 6 Department of Urology, The Jikei University School of Medicine, Tokyo 105-8461, Japan; 7 Department of Kidney Regenerative Medicine, The Jikei University School of Medicine, Tokyo 105-8461, Japan

**Keywords:** organoid, pig, fetus, kidney, cryopreservation

## Abstract

Fetal organs and organoids are important tools for studying organ development. Recently, porcine organs have garnered attention as potential organs for xenotransplantation because of their high degree of similarity to human organs. However, to meet the prompt demand for porcine fetal organs by patients and researchers, effective methods for producing, retrieving, and cryopreserving pig fetuses are indispensable. Therefore, in this study, to collect fetuses for kidney extraction, we employed cesarean sections to preserve the survival and fertility of the mother pig and a method for storing fetal kidneys by long-term cryopreservation. Subsequently, we evaluated the utility of these two methods. We confirmed that the kidneys of pig fetuses retrieved by cesarean section that were cryopreserved for an extended period could resume renal growth when grafted into mice and were capable of forming renal organoids. These results demonstrate the usefulness of long-term cryopreserved fetal pig organs and strongly suggest the effectiveness of our comprehensive system of pig fetus retrieval and fetal organ preservation, thereby highlighting its potential as an accelerator of xenotransplantation research and clinical innovation.

## 1. Introduction

The number of patients requiring kidney transplantation, including those with chronic kidney disease, is increasing worldwide. However, due to the shortage of donors for kidney transplantation, only a small fraction of patients can undergo renal transplantation. Recently, porcine kidneys, similar to human kidneys in size, have gained attention as a potential new source for transplantation. Xenotransplantation studies utilizing porcine kidneys have been actively conducted, including porcine kidney transplantation in brain-dead patients [1]. Nevertheless, improvements are needed, particularly in developing potent immunosuppressive agents.

To overcome this issue, achieving a close resemblance to human organs in transplants is crucial. We are exploring the utilization of an embryonic developmental environment to create alternative chimeric kidneys [2]. In our previous studies, chimeric kidneys with filtration and erythropoietin-expressing abilities were generated by injecting human or rat nephron progenitor cells (NPCs) into mouse metanephroi (MN, also known as metanephric kidneys) [2]. However, due to size limitations in rodents, we are exploring pigs, particularly fetal pigs, as a potential alternative species.

Porcine MNs also offer several other advantages for research, including a greater ability to be cryopreserved due to their smaller size compared to adult-sized organs and their ability to be research sources for fetal kidney organoids. We previously verified the ability of short-term cryopreservation methods to preserve the viability of porcine MNs and reported the reinitiation of kidney development after transplantation of cryopreserved porcine MNs into immunocompromised mice [3]. However, whether cryopreserved porcine MNs can be stored for extended periods remains uncertain. Furthermore, although various organoids are being used in disease models, drug screening, and some clinical cell therapies [4,5,6], the ability of cryopreserved MN to form organoids has not yet been established.

Therefore, in this study, we investigated the efficacy of our comprehensive system for the retrieval and long-term cryopreservation of porcine organs by evaluating the utility of cryopreserved MNs extracted from porcine fetuses obtained through cesarean section without loss of the mother pig’s viability and reproductive capacity. Firstly, we pathologically examined the tissue formation ability of long-term cryopreserved porcine MNs by transplanting them intraperitoneally into immunocompromised mice. Next, we evaluated whether thawed, cryopreserved porcine MNs transported to a remote research facility retained their capacity to form kidney organoids.

## 2. Results

### 2.1. Efficient Retrieval of Pig Fetuses While Preserving Maternal Fertility

**Figure 1:**
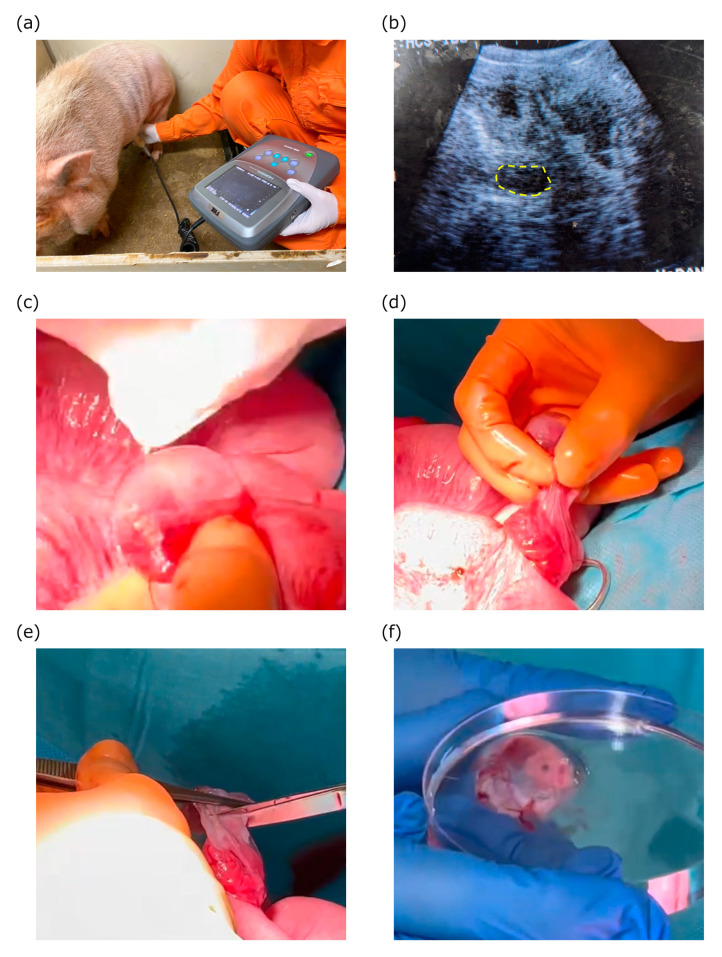
Porcine pregnancy evaluation and cesarean section procedure. (**a**) Pregnancy evaluation. The probe is placed on the pig to check for hydatidiform moles in the uterus. (**b**) An echo image shows a gestational sac (circled). (**c**) The fetus is revealed as extending from the uterus with its amniotic sac intact. (**d**) The amniotic membrane is manually pulled away from the endometrium. (**e**) The amniotic membrane is cut to separate the fetus from the uterus. (**f**) The surgically extracted fetus is wrapped in the amniotic membrane, which is removed in a subsequent step.

We investigated a method of extracting fetuses from pregnant sows to achieve a stable and efficient supply of porcine fetuses. Using an animal ultrasound imaging device, we confirmed the pregnancy of the sows on or after the 23rd day of gestation (Figure 1a,b). On the 29th or 30th day of gestation, the sows were subjected to a cesarean section, and the uterus was removed from the abdominal cavity through a midline incision. Subsequently, the uterine wall was incised to reveal the fetuses enclosed within the amniotic sac (Figure 1c). By gently pulling the fetuses enveloped by the amniotic sac by hand (Figure 1d), we successfully extracted the fetuses in their amniotic-sac-covered state by cutting them off from the placenta using scissors (Figure 1e,f). Using this method, we confirmed that the uterus and peritoneum of the sow could be sutured after fetal extraction, ensuring not only the survival of the sow but also the maintenance of fertility. We performed 31 surgeries, with an average extraction yield of approximately five fetuses per procedure.

### 2.2. Estimation of the Usability of the Cryopreserved Porcine Kidneys by In Vivo Implantation

Table 1 details the characteristics of the seven microminiature pig (MMP) MNs evaluated in this study. The MMP fetuses were retrieved at embryonic days 29 or 30, and the period of kidney sample cryopreservation ranged from 1 day to 2 years. In all samples except one, the post-transplant renal size increased approximately two-fold, further confirming the presence of numerous glomerular structures even after at least 2-year long-term cryopreservation.

The embryonic days, cryopreservation period, growth ratio, and glomerular number for each sample of the in vivo method are shown. The growth ratio represents the posterior kidney’s long diameter before transplantation compared to two weeks post-transplantation. The glomerular number indicates the sum of the number of glomeruli in three sections of the post-transplanted metanephroi.

**Figure 2:**
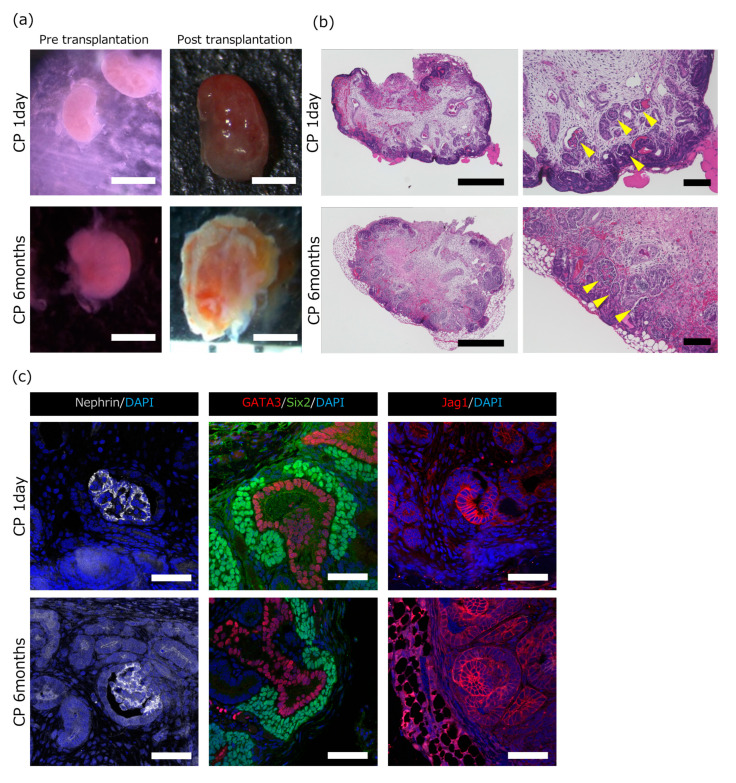
Assessment of the growth and development of the transplanted frozen fetal kidney. Cryopreservation periods were 1 day and 6 months, respectively. (**a**) Macroscopic view of MNs before and after transplantation into adult immunocompromised mice. (**b**) Hematoxylin and eosin staining of the transplanted cryopreserved MNs. Glomeruli are indicated by yellow arrowheads (scale bars: 1 mm in (**a**), 500 μm in (**b**) left, 100 μm in (**b**) right). (**c**) Staining for NEPHRIN (glomerular epithelial cells), sine oculis homeobox homolog 2 (SIX2, nephron progenitor cells), GATA-binding protein 3 (GATA3, ureteric buds and collecting ducts), JAGGED 1 (JAG1, S-shaped bodies and ureteric tubules), and 4′,6-diamidino-2-phenylindole, dihydrochloride (DAPI) (scale bars: 50 μm).

Figure 2 shows bright-field images of pre- and post-transplantation MNs and images of hematoxylin and eosin (HE)-stained and immunostained MNs cryopreserved for 1 day or 6 months. The kidneys subjected to long-term freezing for 6 months demonstrated growth after transplantation similar to that of the kidneys subjected to short-term freezing (Figure 2a). Despite sparse cell density areas within both the short- and long-term frozen sections, the formation of glomerular structures was confirmed in the cortex of the MN (Figure 2b, arrow). Additionally, we evaluated the developmental stages of glomeruli by immunostaining (Figure 2c). Subsequently, a cap structure resembling an overlay of NPCs on ureteric buds (UBs) developed into comma- and S-shaped bodies that eventually become glomeruli [7]. In both cryopreserved samples, NEPHRIN-positive glomeruli were observed, indicating that renal maturation was achieved by transplantation into immunocompromised mice after cryopreservation (Figure 2c, left). Cap structures were identified by the presence of SIX2-positive and GATA3-positive cells, and S-shaped structures were identified by the presence of JAG1-positive cells (Figure 2c, center and right). This result indicated the presence of glomerular structures at various stages of maturation, even after long-term cryopreservation.

### 2.3. Successful Production of Pig Renal Organoids from the Cryopreserved Fetal Kidneys

**Figure 3:**
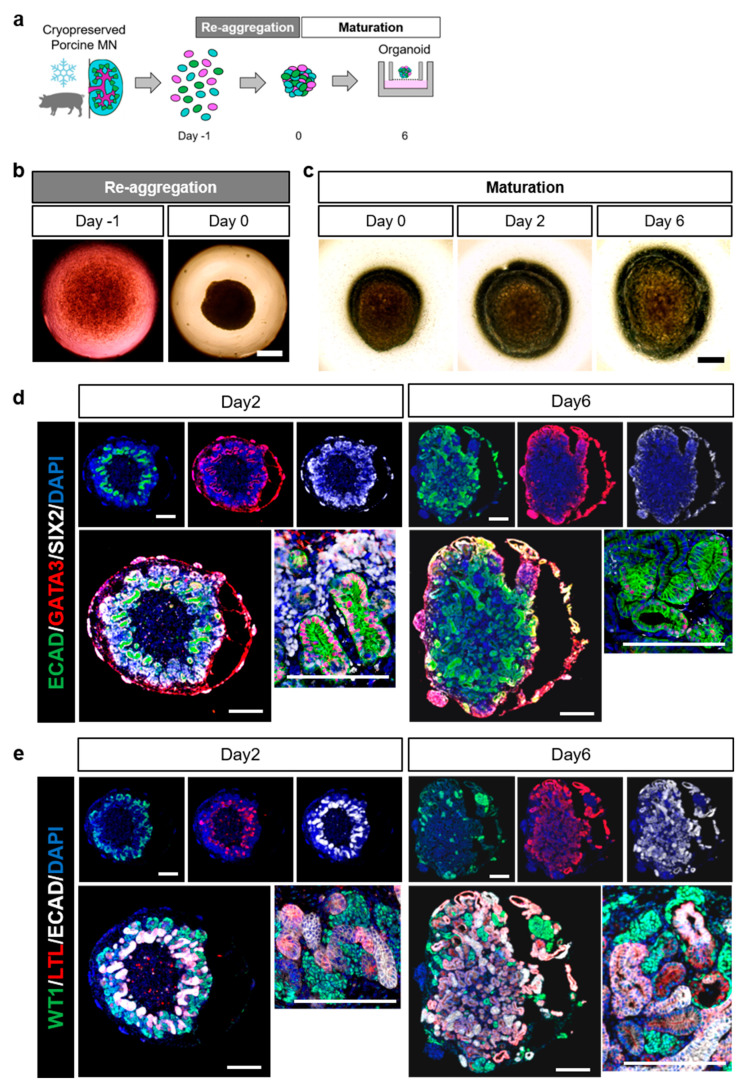
Assessment of the organoid formation and maturation from frozen porcine fetal kidneys. (**a**) Schematic representation of organoid culture from cryopreserved pig metanephric kidneys (MNs). (**b**) Representative bright-field images of single-cell dissociated MNs at day −1 and reaggregated MNs at day 0 (scale bar: 500 μm). (**c**) Representative bright-field images of porcine fetal kidney organoids cultured at the air–liquid interface at days 0, 2, and 6 (scale bar: 500 μm). (**d**) Representative images of day 2- and day 6-cultured porcine fetal kidney organoids stained with anti-E-cadherin-1 (ECAD, distal tubules), anti-GATA-binding protein 3 (GATA3, ureteric buds and collecting ducts), anti-sine oculis homeobox homolog 2 (SIX2, nephron progenitor cells), and 4′,6-diamidino-2-phenylindole, dihydrochloride (DAPI) (scale bar: 500 μm). (**e**) Representative images of day 2- and day 6-cultured pig MN organoids stained with anti-Wilms’ tumor-1 (WT1, glomeruli), anti-lotus tetragonolobus lectin (LTL, proximal tubules), anti-ECAD (distal tubules), and DAPI (scale bar: 500 μm).

To investigate whether the frozen MNs formed organoids, MMP MNs cryopreserved for 1 month were dissociated into single cells and cultured in a reaggregation medium for 24 h to form spherical aggregates (Figure 3b). Subsequent culturing of the spheroids at the air–liquid interface revealed the gradual expansion of the organoid structure over time (Figure 3c). Immunostaining with representative NPC markers (SIX2), nephron segment markers (WT1, LTL, and ECAD), and UB markers (GATA3) (Figure 3d,e) indicated that organoids were rich in SIX2-positive nephron progenitor cells on day 2, displaying multiple cap-like structures at the early stages of renal development (Figure 3d,e, left). By day 6, the SIX2-positive NPCs had almost disappeared in the organoids and instead had differentiated and matured into glomeruli, proximal tubules, distal tubules, UBs, and their progenitor cells (Figure 3d,e, right). These data indicate that cryopreserved porcine MNs can survive and resume development and differentiation after thawing, strongly suggesting the usefulness of porcine MNs as cryopreserved organs.

## 3. Discussion

The best treatment for end-stage renal failure is kidney transplantation; however, few people can benefit from it due to a severe shortage of donors. Recently, xenotransplantation of porcine kidneys as an alternative to human kidneys has attracted attention. However, despite advances in genetic engineering, concerns about strong immunosuppressive agents have not yet been fully addressed. Our research group is undertaking the challenge of “regenerative medicine using porcine fetuses for xenotransplantation”, aiming to generate transplantable kidneys by injecting human iPSC-derived NPCs into porcine MNs for organogenesis [2]. This approach has advantages over conventional xenotransplantation of adult organs. First, our approach overcomes the challenges of immune rejection. Our method of using chimeric kidneys composed of transplanted human cells to replace the major constituent cells of the kidney has significant immunological advantages over conventional xenotransplantation methods [2]. Another advantage is the use of cryopreserved MNs. As demonstrated in this study, porcine MNs can be cryopreserved even for extended periods, and they are transportable because of their small size [3]. This characteristic enables the rapid supply of organs to meet the demands of patients and researchers. Additionally, this study provides evidence that porcine fetal kidneys obtained through cesarean section can be effectively retrieved, cryopreserved, and utilized for an extended period, suggesting that fetal organs can be obtained and preserved for future use, even in pigs with limited breeding numbers, such as those intended for clinical applications. Therefore, the realization of a novel method of renal organ regeneration utilizing fetal porcine kidneys and its clinical application holds significant potential for the future.

In this study, we confirmed that porcine MNs can be preserved for 2 years using proper storage procedures. Cell cryopreservation studies have shown that canine and human adipose tissues, platelets, pancreatic cells [8,9,10,11,12], and even small organs, such as rat kidneys, can be cryopreserved [13]. Although intracellular ice crystal formation presents a challenge in the cryopreservation process, it can be averted by adequate immersion in a suitable freezing solution and replacement of water content. Cryopreservation of relatively large organs is difficult because the freezing solution cannot effectively penetrate deep into the organs. Even if xenotransplantation were to advance in the future, cryopreservation of transplanted adult porcine kidneys would remain a substantial challenge because porcine kidneys are significantly larger than rat kidneys. However, because the MNBs of the porcine fetus are smaller than those of adult porcine kidneys, cryopreserving them without disrupting the organ structure is feasible. Various kidney cells, including NPCs, UBs, and stromal cells, interact via signaling pathways to establish the structural regularity of the kidney [7]. In this study, we directly cryopreserved intact porcine MNBs, ensuring the presence of all renal cell types necessary for development and preserving the structural regularity of the kidney. These factors contribute to the successful resumption of appropriate renal development even after thawing. Previous reports have compared canine mesenchymal stem cells stored in cryopreservation solution for approximately 1 year with those stored unfrozen and showed comparable survival rates, cell markers, and functionality [11]. In humans, the viability and functionality of platelets frozen in sheet form for 18 months do not differ from those of non-frozen platelets [12]. These results are consistent with our results, indicating that the cells retain viability, functionality, and differentiation capacity after cryopreservation.

Furthermore, we succeeded in producing fetal porcine kidney organoids using cryopreserved cells. In humans, stem cell organoids are used in disease models, drug screening, and clinical cell therapies [4,5,6]. Recently, porcine kidney organoids derived from porcine naïve embryonic stem cells have been reported [14]. However, cells in kidney organoids are extremely immature, and current technology does not support the in vitro induction of all the cells that make up the kidney [15,16,17]. Conversely, organs removed from animal fetuses form organoids [18]. These results suggest that porcine MNs can be appropriately cryopreserved to maintain their viability and developmental potential. Because pigs are more similar to humans than rodents regarding the gestation period and organ size, porcine organoids may serve as valuable tools for accelerating research on organ development. Moreover, owing to the limited availability of pig breeding facilities, the research or medical use of pigs and their organs poses challenges regarding accessibility and high costs. This study establishes a method for obtaining porcine fetuses without loss of the mother pig’s fertility and demonstrates the cryopreservation capability of porcine MNs, which significantly contributes to the effective utilization of such valuable resources.

This study had two limitations. First, variations in glomerular counts were observed in the pathological examination for unknown reasons. Despite being removed and stored from the same fetus, one MN had a low number of glomeruli, and the other had many glomeruli that developed after transplantation (6 months of cryopreservation period; Table 1). The MNs were cryopreserved using the same freezing solution and procedures. The difference might depend on where the MN was transplanted, the degree of bleeding around the transplantation area, the amount of blood vessels from the host, or the general condition of the host. However, the precise cause remains unidentified and warrants further investigation. Furthermore, more mature organs can be obtained by the addition of growth factors. Second, sparse cell density areas were observed in the interior of the MNs (Figure 2b). We speculate that this is due to the fact that the environment in which the MNs are placed after transplantation is not necessarily the same as the conventional developmental environment. During this developmental stage, MNs are expected to extensively incorporate host blood vessels immediately; however, the initiation of host blood vessel incorporation occurred after transplantation, potentially leading to transient ischemic conditions that could have caused delayed MN development. We also suggest that our procedure may cause delayed urine production and/or tubular growth retardation. These factors may have caused a decrease in the cell density in the area where the tubules would have grown. Therefore, better evaluation methods and further investigations are required.

In conclusion, our study demonstrated that MNs of porcine fetuses obtained through cesarean section without loss of the mother pig’s reproductive capacity could be cryopreserved for up to 2 years. Additionally, we successfully generated organoids from cryopreserved porcine fetal kidneys. These results underscore the utility of long-term storage of porcine organs by cryopreservation and strongly indicate the effectiveness of our comprehensive system of porcine fetus retrieval and subsequent organ preservation. As research progresses and porcine MNs or their derivatives become viable for clinical transplantation, they offer a promising solution for patient-centered therapies, enabling long-term storage independent of transplant logistics. Additionally, the successful development of porcine kidney organoids holds promise for advancing mammalian kidney research and drug screening endeavors.

## 4. Materials and Methods

### 4.1. Research Animals

All the animals were treated in accordance with the Guidelines for the Proper Conduct of Animal Experiments. The animal studies were approved by the Animal Ethics Committee of Jikei University School of Medicine (approval numbers: 2021-069 and 2020-081). Pregnant MMPs (Fuji Micra, Inc., Shizuoka, Japan) were used to extract MNs connected to the bladders (MNBs) from fetal pigs. Male immunocompromised mice (NOD/Shi-scid, IL-2RgKO Jic mice; CLEA Japan Inc., Tokyo, Japan) were used as recipients.

### 4.2. Cesarean Section and Fertility Preservation in Pigs

Pregnant MMPs were selected from sows that had undergone vaginal delivery, and the day after mating was considered embryonic day 0 (E0). Pregnancy was determined using a veterinary ultrasound imaging device (HS-101V; Honda Electronics Co., Ltd., Aichi, Japan) at approximately E23 (embryonic day 23; Figure 1a,b). In this study, cesarean sections were performed up to twice, and vaginal delivery was allowed in pregnant pigs.

### 4.3. Collection, Cryopreservation, and Thawing of Fetal Porcine Kidneys

Procedures were performed as previously described [3]. In brief, a cesarean section was performed on pregnant MMPs on E29 or E30, and the fetuses were transported in carbon dioxide (CO2)-equilibrated minimum essential medium (MEM-α; 12561-056; Invitrogen, Waltham, MA, USA) on ice to another laboratory. MNBs were extracted as tissue blocks and equilibrated in base medium (MEM-α supplemented with 20% fetal bovine serum [FBS; SH30070.03, HyClone Laboratories, Inc., Logan, UT, USA], and 0.5% Penicillin-Streptomycin [168-23191; FUJIFILM Wako Pure Chemical Corporation, Osaka, Japan]) with 7.5% dimethyl sulfoxide (DMSO; 317275-100ML; Millipore, Burlington, MA, USA) and 7.5% ethylene glycol (EG; 055-00996; FUJIFILM Wako Pure Chemical Corporation) on ice for 15 min and soaked in base medium with 15% DMSO and 15% EG on ice for an additional 15 min. The MNBs were placed on Cryotops (81111; Kitazato Corporation, Tokyo, Japan) and directly plunged into liquid nitrogen. They were stored in liquid nitrogen tanks until used for experiments. To thaw porcine MNs, Cryotops with MNBs were quickly transferred to the base medium with 1 M sucrose at 42 °C for 1 min, transferred to the base medium with 0.5 M sucrose at room temperature for 3 min, and finally washed twice in the base medium at room temperature for 5 min each time. The MNs were separated from the bladder and trimmed under a stereomicroscope.

### 4.4. In Vivo Fetal Kidney Transplantation Assay

Procedures were performed as previously described [3]. Immunocompromised mice were subjected to a laparotomy under isoflurane inhalation anesthesia. A pocket was created in the retroperitoneum in the area bounded by the aorta, left ureter, and left renal artery. One MN per recipient was transplanted into the pocket, which was then closed. Two weeks after transplantation, the grafts were harvested. The lengths of the grafts were measured before and after transplantation, and the growth ratio was calculated. Harvested kidneys were fixed in 4% paraformaldehyde (161-20141; FUJIFILM Wako Pure Chemical Corporation) at 4 °C overnight and embedded in paraffin. Long-axis sections with a thickness of 5 μm were prepared from the whole kidneys. Three sections from each of the first, median, and third quartiles of thickness were selected for HE staining using standard procedures. For immunostaining, slides with affixed paraffin sections were deparaffinized, incubated with citrate buffer (#K035; Cosmo Bio Co., Ltd. Tokyo, Japan) at 121 °C for 10 min for antigen retrieval, washed thrice in PBS-T, blocked at room temperature for 10 min using Blocking One Histo (06349-64; Nacalai Tesque, Inc., Kyoto, Japan), washed thrice in PBS-T, and incubated overnight at 4 °C with primary antibodies. On the next day, after washing thrice in PBS-T, the slides were incubated with secondary antibodies at room temperature for 1 h and mounted using SlowFade Diamond Antifade Mountant (P36963; Thermo Fisher Scientific, Inc., Waltham, MA, USA). Regarding the primary antibodies, we used anti-sine oculis homeobox homolog 2 (SIX2; 11562-1-AP; Thermo Fisher Scientific, Inc.) for NPCs, anti-GATA-binding protein 3 (GATA3; AF2605; R&D Systems, Inc., McKinley, MN, USA) for UBs, anti-NEPHRIN (GP-N2; PROGEN Biotechnik GmbH, Heidelberg, Germany) for glomeruli, and anti-JAGGED 1 (JAG1; sc-6011; Santa Cruz Biotechnology, Inc., Dallas, TX, USA) for S-shaped bodies. Regarding the secondary antibodies, we used Alexa Fluor 488 donkey anti-rabbit immunoglobulin G (IgG) antibody (A21206; Thermo Fisher Scientific, Inc.), Alexa Fluor 555 donkey anti-goat IgG antibody (A21432; Thermo Fisher Scientific, Inc.), and Alexa Fluor 647 donkey anti-guinea pig IgG antibody (706-605-148; Jackson ImmunoResearch Inc., West Grove, PA, USA). We used 4′,6-diamidino-2-phenylindole, dihydrochloride (DAPI) for nuclear staining (D1306; Invitrogen). All samples were evaluated under a fluorescence microscope (LSM880; Carl Zeiss AG, Oberkochen, Germany).

### 4.5. In Vitro Organoid Experiment Assay

The cryopreserved pig MNs were placed in a cryoshipper (SC2/1V; Bio Medical Science, Inc., Tokyo, Japan) with liquid nitrogen, and the appropriate procedures were performed at Jikei University School of Medicine to Sumitomo Pharma Co., Ltd. Immediately after thawing, the porcine MNs were collected in 1.5 mL tubes containing MEM-α. Porcine MN dissociation into single cells was performed as below. Firstly, the 1.5 mL tubes were centrifuged at 700× g for 3 min. The supernatants were removed, and 1 mL of room-temperature Accutase (AT104; Innovative Cell Technologies, Inc., San Diego, CA, USA) was added. After vortexing the mixture for 30 s and incubating it at 37 °C for 5 min, the same procedure was repeated twice. The mixture was successively pipetted for MN dissociation and incubated again at 37 °C for 5 min. The same procedure was repeated twice. After centrifugation at 300× g for 5 min and removal of the supernatants containing accutase, 1000 μL of PBS with 10 μM Y27632 (034-24024; FUJIFILM Wako Pure Chemical Corporation) was added to the pellets, single-cell suspensions were prepared by passage through a 40 µm cell strainer (352340: BD Falcon, Oxford, UK), and the cells were counted to adjust the concentration to 10^6^ cells/mL in basic medium [19] with 1 μM CHIR99021 (038-24681; FUJIFILM Wako Pure Chemical Corporation), 5 ng/mL FGF-9 (ab256004; Abcam plc., Cambridge, UK), and 10 μM Y27632 (034-24024; FUJIFILM Wako Pure Chemical Corporation). The cell suspension was added to V-bottom 96-well plates (MS-9096V; Sumitomo Bakelite Co., Ltd., Osaka, Japan) at 2 × 10^5^ cells/well. Finally, the cell-seeded 96-well plates were centrifuged at 1000 rpm for 4 min, incubated in a 37 °C incubator for 24 h, and cultured in an air–liquid interface environment for another 48 h in basic medium supplemented with 1 µM CHIR99021 (038-24681; FUJIFILM Wako Pure Chemical Corporation) and 10 ng/mL human FGF-9 (ab256004; Abcam plc.). Following the replacement of the medium with basic medium supplemented with 10 ng/mL human FGF-9 and incubation for another 4 days, the samples were harvested and fixed overnight at 4 °C with 4% PFA. After the fixative was replaced with 30% sucrose, the samples were stored at 4 °C until they settled, then they were cryo-embedded using an embedding compound (3801481; Leica Biosystems Nussloch GmbH, Wetzlar, Germany), sectioned to a thickness of 10 μm near the center for immunostaining, and mounted onto slides. The slides were washed with TBS-T, incubated with antigen retrieval reagent (RM102-H; LSI Medience Corporation, Tokyo, Japan) at 121 °C for 5 min, washed in TBS-T, blocked at room temperature for 60 min using PBS supplemented with 2% normal donkey serum (IHR-8135; ImmunoBioScience Corp, Mukilteo, WA, USA) and 0.3% Triton X-100 (35501-15; Nacalai Tesque, Inc.), and incubated overnight at 4 °C with primary antibodies. On the next day, the slides were incubated with secondary antibodies at room temperature for 1 h and mounted using FluorSave Reagent (345789; Millipore). Regarding the primary antibodies, we used anti-Wilms’ tumor-1 (WT1; ab89901, Abcam plc.) for the glomerulus, anti-lotus tetragonolobus lectin (LTL; B-1325; Vector Laboratories Inc., Burlingame, CA, USA) for the proximal tubules, anti-E-cadherin-1 (ECAD; 610181, BD Biosciences, San Jose, CA, USA) for the distal tubules, anti-GATA3 (AF2605; R&D Systems, Inc. for UB, and anti-SIX2 (11562-1-AP; Thermo Fisher Scientific, Inc.) for NPCs. Regarding the secondary antibodies, we used Alexa Fluor 488 donkey anti-rabbit IgG antibody (A21206; Thermo Fisher Scientific, Inc.), Alexa Fluor 488 donkey anti-mouse IgG antibody (A21202; Thermo Fisher Scientific, Inc.), Streptavidin, Alexa Fluor 568 donkey conjugate antibody (S11226; Thermo Fisher Scientific, Inc.), Alexa Fluor 594 donkey anti-goat IgG antibody (A11058; Thermo Fisher Scientific, Inc.), Alexa Fluor 647 donkey anti-mouse IgG antibody (A31571; Thermo Fisher Scientific, Inc.), and Alexa Fluor 647 donkey anti-rabbit IgG antibody (A31573; Thermo Fisher Scientific, Inc.). For nuclear staining, we used DAPI (D1306; Invitrogen). All the samples were evaluated under a fluorescence microscope (IN Cell Analyzer 6000; Cytiva, Tokyo, Japan).

## Figures and Tables

**Table 1:** Sample details of pig fetal metanephric kidneys from the in vivo method.

Embryonic Days	Cryopreservation Period	Growth Ratio	Glomerular Number
30	1 day	2.20	13
30	2 days	1.87	38
30	3 days	1.90	23
30	6 months	1.73	32
30	6 months	1.14	2
29	2 years	2.46	63
29	2 years	1.90	26

## Data Availability

The datasets generated or analyzed in the current study are available from the corresponding author upon reasonable request.
